# Molecular Orientation Behavior of Lyotropic Liquid Crystal–Carbon Dot Hybrids in Microfluidic Confinement

**DOI:** 10.3390/ijms25105520

**Published:** 2024-05-18

**Authors:** Artem Bezrukov, Aliya Galeeva, Aleksandr Krupin, Yuriy Galyametdinov

**Affiliations:** Department of Physical and Colloid Chemistry, Kazan National Research Technological University, 68 Karl Marx Str., 420015 Kazan, Russia; galeeva-alija@mail.ru (A.G.); krupin_91@mail.ru (A.K.); yugal2002@mail.ru (Y.G.)

**Keywords:** lyotropic liquid crystals, carbon dots, quantum dots, microfluidics, molecular ordering, lamellar structures

## Abstract

Lyotropic liquid crystals represent an important class of anisotropic colloid systems. Their integration with optically active nanoparticles can provide us with responsive luminescent media that offer new fundamental and applied solutions for biomedicine. This paper analyzes the molecular-level behavior of such composites represented by tetraethylene glycol monododecyl ether and nanoscale carbon dots in microfluidic channels. Microfluidic confinement allows for simultaneously applying multiple factors, such as flow dynamics, wall effects, and temperature, for the precise control of the molecular arrangement in such composites and their resulting optical properties. The microfluidic behavior of composites was characterized by a set of analytical and modeling tools such as polarized and fluorescent microscopy, dynamic light scattering, and fluorescent spectroscopy, as well as image processing in Matlab. The composites were shown to form tunable anisotropic intermolecular structures in microchannels with several levels of molecular ordering. A predominant lamellar structure of the composites was found to undergo additional ordering with respect to the microchannel axis and walls. Such an alignment was controlled by applying shear and temperature factors to the microfluidic environment. The revealed molecular behavior of the composite may contribute to the synthesis of hybrid organized media capable of polarized luminescence for on-chip diagnostics and biomimetics.

## 1. Introduction

Organized anisotropic media represented by liquid crystal (LC) molecular materials attract a sustainable and growing research interest supported by complementary research fields such as bioimaging, sensing, and microfluidics [1,2,3]. The selective orientation sensitivity of liquid crystal molecules to delicate changes in internal and external conditions makes both thermotropic and lyotropic liquid crystals (LLC) convenient for applications in temperature and pH sensing [4], security labeling [5], biomedical analysis [6,7,8], and the quantitative analysis of various chemical and biochemical agents [9,10,11].

The modification of liquid crystals with luminescent nanoparticles is an approach to creating media with polarized luminescence capabilities [1,12]. The benefits of polarized luminescence from ordered molecular materials for photonics and imaging attracted mature research attention [13,14] and recent interest [12,15,16], with a specific trend of incorporating nanoparticles with bioimaging potential, such as quantum dots [17,18], into an LC matrix [19,20].

Liquid crystals and their composites with various dopants allow for conveniently tracking molecular-level processes such as the aligning and anchoring of LC molecules by microscale changes in the optical behavior of LC media. In this respect, microfluidic channels represent a particularly attractive microscale non-equilibrium environment for characterizing and tuning the orientation and optical behavior of such materials [21,22]. A systematic study of thermotropic microfluidic LC systems (mostly nematic) started in the last decade [2,23,24,25]. Microchannels provide new factors that control the orientation of LC molecules, such as anchoring to channel walls and responding to a finely tunable shear [23,26,27]. Microchannel confinement is a suitable environment for the convenient generation of LC dispersions [10,11,28]. Microfluidic devices with an integrated LC matrix are rapidly developing as convenient laboratory-on-chip instruments [4,28,29,30,31].

As compared with thermotropic liquid crystals, the advantages of microfluidics for characterizing the behavior of lyotropic liquid crystals also attracted attention in the last decade [32,33]. Related research activities are more fundamental and recent and are mostly represented by publications of the last several years [34,35,36,37,38,39], where authors discussed the general aspects of orientation and the phase behavior of confined LLC systems. An interesting feature of LLC in microchannels is that they can be convenient models of biological anisotropic liquids in living capillary systems [40,41]. Coupling LLC systems with nanoscale carbon dots and characterizing such composites in non-confined conditions are discussed in resent research reports [19,42,43], while the microfluidic behavior of such hybrids is expecting a focused and systematic investigation.

This work aims at studying the molecular orientation and related optical behavior of anisotropic luminescent media represented by composites of a lyotropic liquid crystal with nanoscale carbon dots. This study integrates and continues our previous research on lyotropic liquid crystals [44,45] and microfluidic optically active media [46,47]. We characterized the molecular orientation responses of static composites to both external and intrinsic microfluidic factors such as heating and cooling and wall effects. We analyzed the response of the LLC media to shear in dynamic microflows and correlate polarized light transmission intensity with ongoing molecular-level processes. Finally, we evaluated the specific impact of doped carbon dots on the molecular alignment of the LLC matrix in various conditions and analyzed the luminescence properties of the composites.

## 2. Results

### 2.1. Preliminary Characterization of the LLC and Composite

**Figure 1:**
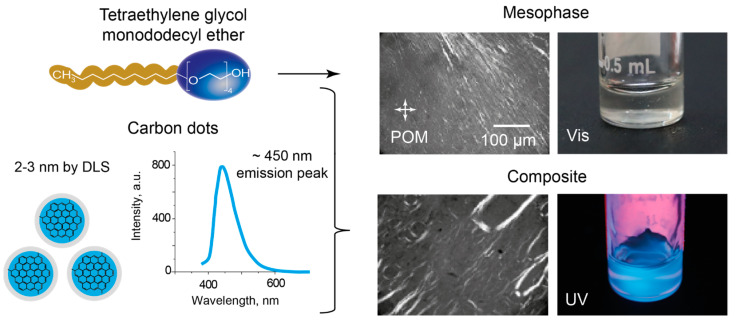
Schematic structures of tetraethylene glycol monododecyl ether and carbon dots, polarized microscopy images of the lyotropic mesophase and its composite with carbon dots, and their macroscopic photos in visible and UV light, respectively.

An important requirement for an optically active material suitable for molecular diagnostic and lab-on-chip applications is its ability to maintain useful properties at the standard temperature of 25 °C and/or in the physiological temperature range. On the other hand, a convenient research selection can be a lyotropic liquid crystal with a previously characterized structure of a mesophase that may facilitate discussing the behavior of LLC and its composite with carbon dots in microfluidic confinement. In this work, we studied the composite of blue luminescent carbon dots (bCD) and tetraethylene glycol monododecyl ether C_12_EO_4_, which represents a well-characterized compound exhibiting lyotropic mesomorphism in the temperature range mentioned above. The components of the composite were studied by polarized optical microscopy (POM), dynamic light scattering, and fluorescence spectroscopy. The results are summarized in Figure 1.

Tetraethylene glycol monododecyl ether molecules form nonionic surfactant systems. Their molecular structure demonstrates a large size ratio of polar head groups to nonpolar hydrophobic tails. Such molecules tend to form lamellar surfactant assemblies [48] that maintain their lamellar structure upon the addition of various dopants, as was shown in our previous work [49], through X-ray diffraction. Lamellar layers were shown to form internal hydrophobic areas with interpenetrating hydrocarbon tails [50].

The carbon dots that were used in this work are nanoparticles with an approximate size of 2–3 nm, according to our characterization in dynamic light scattering experiments. The DLS size distribution curve is provided in Supplementary Materials, Figures S1–S4. The emission peak of the C-dots is observed at ~450 nm. These C-dots were synthesized by a hydrothermal method [51] and are able to form aqueous dispersions. The size of the synthesized C-dots agrees with the results represented in the literature, where authors performed the size characterization of C-dots synthesized from similar precursors by DLS [52,53,54] and transmission electron microscopy [51,55,56].

The POM images of both the individual LLC and the composite (Figure 1) show characteristic patterns observed for lamellar lyotropic mesophases [57]. Experimental evidence indicating that C_12_EO_4_ molecules are capable of forming a lamellar mesophase was demonstrated in our previous work through X-ray diffraction studies [49]. Diffractograms of the C_12_EO_4_ phase showed patterns typical for a lamellar organization of LLC molecules. Such an organization remained stable upon the introduction of luminescent additives. Similar polarized microscopy patterns of the basic LLC and the composite indicate a similar intrinsic organization of the LLC phase in the basic liquid crystal and the composite.

According to X-ray diffraction studies, such a lamellar system included interplanar regions with thicknesses of about 5–6 nm. Such interplanar areas, which are located between lamellae with internal hydrophobic regions, can be a potential hosting environment for water-soluble C-dots.

In visible light, both LLC and its composite are transparent fluids. The basic LLC was insensitive to macroscopic UV excitation conditions, while the composite demonstrated bright blue light luminescence (Figure 1).

**Figure 2:**
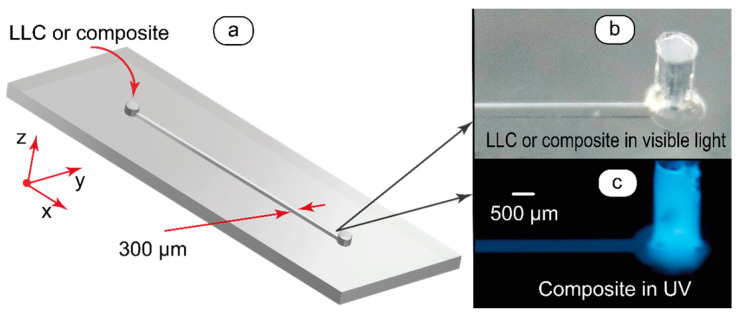
Design of microfluidic devices and images of the lyotropic mesophase and its composite with carbon dots taken in visible and UV light, respectively: (**a**) microchannel geometry, (**b**,**c**) macrophotography imaging of the microchip output in visible and UV light, respectively. Red arrows show the coordinate axes.

Microfluidic devices with rectangular 100 µm × 300 µm (height × width) microchannels were used in this work (Figure 2). The chip design is shown in Figure 2a. According to our previous works [46,47], such microchannels (100–300 µm wide) allow for obtaining high-quality POM images with easily distinguishable patterns of LC textures.

Macrophotography imaging of microfluidic channels filled with composite allowed for directly observing the luminescence of the composite in the microchannel. In visible light (Figure 2b), the composite was transparent and could not be distinguished on the background of the transparent microchip material that is polydimethylsiloxane (PDMS). The luminescence of the composite (Figure 2c) was easily distinguishable at the sub-macroscale level both in the chip output and the microchannel itself. A high-contrast channel image in UV indicated no notable penetration of the composite into bulk PDMS.

Thus, pure LLC and LLC-bCD composites showed similar textures in polarized light, which were observed for the lamellar organization of LLC molecules and previously confirmed for the basic LLC system. The composite demonstrated intensive luminescence and was compatible with PDMS microfluidic chips. At the next stages of this work, we focused on characterizing the behavior of LLC and LLC-bCD composites in microfluidic confinement.

### 2.2. Molecular Orientation and Optical Properties of Static LLC and Carbon Dot Composites in Microchannels

**Figure 3:**
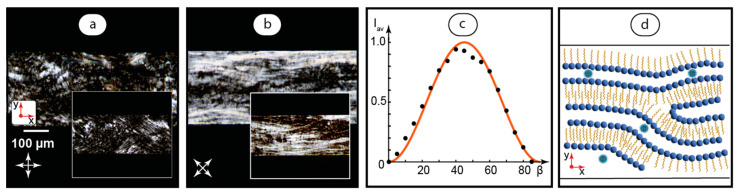
Static samples of the composite infused into a microchannel: (**a**,**b**) POM images of the composite and reference samples of basic LLC (insets); (**c**) normalized average transmission intensity vs. the angle of the polarizer with respect to the microchannel axes (dots) and its theoretical values (solid curve); (**d**) suggested orientation of mesophase molecules in the composite and presumable arrangement of C-dots. Red arrows represent coordinate axes with respect to Figure 2. Crossed arrows indicate the positions of polarizers.

The first step of studying the composites in microfluidic confinement focused on their behavior in static conditions. Before the experiments, LLC systems were infused into microchannels with a flow velocity of about 1 mm/s and then kept static. Figure 3 shows their polarized microscopy images and the presumable orientation of LLC molecules in the composite.

Figure 3a demonstrates the microfluidic texture of the static composite. This pattern is similar to the texture observed for a static lamellar C_12_EO_4_ mesophase in earlier shear studies [58]. The reference texture of the basic LLC (Figure 3a inset) indicates that the LLC phase undergoes no significant changes after adding C-dots (0.1 g/L) to the initial aqueous phase.

An important POM technique that allows for revealing molecular ordering in LC systems is the rotation of crossed polarizers [59]. The intensity of light transmission through media between crossed polarizers depends on the angle between the director and the polarizer:

(1) $$\frac{I}{I_{0}}\sim {sin}^{2}2\varphi$$

where I and I_0_ are the transmitted and initial light intensities, respectively, and φ is the angle between the polarizer and the director.

In Figure 3a, the polarizer is parallel to the microchannel axis and the analyzer is perpendicular to it. In Figure 3b, both the polarizer and analyzer are rotated 45°, and we can see a significant change in the texture and transmitted light intensity. Such a behavior is different from that of the non-microfluidic textures in Figure 1, which showed no dependence of the light transmission pattern on the rotation of crossed polarizers. It may indicate, therefore, a predominant ordering of LLC molecules in microfluidic samples in a certain direction.

To quantify the orientation of LLC molecules in confinement, we analyzed the transmission intensities of microfluidic samples at various rotation angles of polarizers by taking their POM images and processing them with MATLAB according to the methodology developed in our previous work [47]. According to this methodology, the dimmest and brightest microfluidic images are taken as references, and the normalized average brightness of microchannel POM images is calculated with respect to these references:

(2) $$I_{av}=\frac{\bar{Y}-{\bar{Y}}_{min}}{{\bar{Y}}_{max}-{\bar{Y}}_{min}}$$

where $\bar{Y}$, ${\bar{Y}}_{min}$, and ${\bar{Y}}_{max}$ represent the average brightness of the current, dimmest, and brightest images, respectively.

As the brightness of images is proportional to the transmitted light intensity, we obtain the normalized transmitted light intensities of all the samples in the [0, 1] range (Figure 3c). We can see that the maximum normalized transmittance (black dots) is observed for the rotation angle of about 45°. It corresponds to the theoretical values calculated for LC molecules oriented perpendicular to microchannel walls (solid red curve). The patterns in Figure 3a,b are, however, non-homogeneous, and the average transmittance was used for calculations, so we can assume only a predominant orientation of LLC molecules in a microchannel with certain fluctuations from the director. The respective presumable alignment of LC molecules is shown in Figure 3d, suggesting an additional ordering of lamellae along the microchannel axis. Such an effect may be caused by an initial shear of the LLC phase during its infusion into a microchannel.

Therefore, the second degree of ordering (the axial arrangement of lamellae) appears in microfluidic shear conditions in addition to an intrinsic lamellar arrangement of the LC molecules.

**Figure 4:**
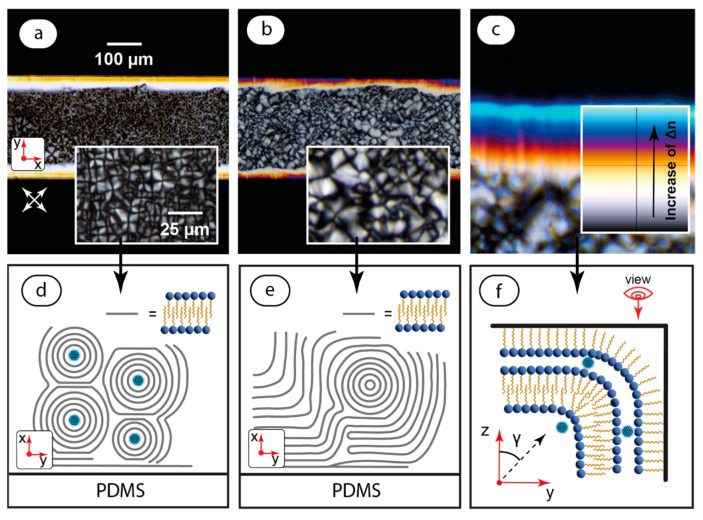
Static samples of the composite in a microchannel after its heating up to the clearing point and further cooling down to 25 °C: (**a**) POM image of the composite and enhanced image of the texture in the middle of the microchannel (inset); (**b**) POM image of the basic LLC and enhanced image of the texture in the middle of the microchannel (inset); (**c**) LLC image at the microchannel edge and its comparison with a Michel–Levy chart (inset); (**d**) a presumable orientation pattern of mesophase molecules in the composite; (**e**) a presumable orientation pattern of mesophase molecules in the basic LLC; (**f**) a presumable orientation pattern of mesophase molecules at the microchannel edge. Arrangement of C-dots is presumable. Red arrows represent coordinate axes with respect to Figure 2. Crossed arrows indicate the positions of polarizers.

At the next stage of characterizing static basic LLC and the composites in microchannels, they were heated above their clearing temperature (over 50 °C) to initiate a complete phase transition to isotropic liquid and then cooled down (2 °C/min) to 25° C. Such a procedure was performed to eliminate aligning effects from an initial injection and enhance a possible impact of microchannel walls on the LLC orientation behavior. The addition of C-dots did not drastically change phase transition temperatures, which can also indicate the formation of a physical mixture without chemical bonding. The results are summarized in Figure 4.

The textures shown in Figure 4 are considerably different from those represented in Figure 3. The microchannel with the infused composite (Figure 4a) contains densely packed cross-like textures in its central part and bright 40–50 µm-wide stripes at its edges. The analysis of the light transmittance by Equation (2) revealed that it does not depend on the rotation angle of polarizers in the central part and corresponds to the plot shown in Figure 2c at microchannel sides. For thermotropic nematic liquid crystals, such an optical behavior indicates the homeotropic orientation of molecules in microchannels [2,23]. A detailed analysis of the enhanced LLC image of the microchannel central part (Figure 4a inset), however, revealed an additional ordering, which was previously reported as lamellar “onions” [60,61,62] or multilayer lamellar structures arranged around certain centers of symmetry.

A similar texture was observed for the basic LLC phase (Figure 4b), although the central pattern was more disordered and “onions” were less distinguishable. Microfluidic samples of both the basic LLC and composite left for 2–3 h after cooling to equilibrate (Figure 4c) reveal characteristic birefringence color patterns at their sides. These patterns coincided with the respective segment of the Michel–Levy chart [63]. This chart can be used to evaluate tilt angles of LC molecules with respect to the vertical axis “z” [64]:

(3) $$\Delta n=\frac{n_{∥}n_{⟂}}{\sqrt{{n_{⟂}}^{2}{sin}^{2}\gamma +{n_{∥}}^{2}{cos}^{2}\gamma }}-n_{⟂}$$

where Δn is birefringence, $n_{∥}$ and n_⟂_ are anisotropic refraction indices, and γ is the tilt angle.

According to Equation (3), the tilt angle increases with birefringence, which can be evaluated from the Michel–Levy chart. Although the accurate evaluation of birefringence requires additional values of the anisotropic refraction indices and the thickness of the birefringent layer at the microchannel edge, we can qualitatively conclude from Figure 4c that the tilt angle increases closer to the side of the microchannel.

Figure 4d–f show LLC textures estimated from POM studies. More ordered “onion” patterns in the composite (Figure 4d) indicate that carbon dots can play the role of central defects that favor the formation of such “onions” around them. In the basic LLC phase, the arrangement of lamellae is assumed to be more disordered (Figure 4f), and the second-order structure is less distinguishable. The birefringence pattern at channel edges indicates that LLC molecules tilt in this section with a suggested bending of lamellar layers to keep LLC molecules perpendicular to microchannel walls. Such patterns were found to be similar both in the composite and the basic LLC, so orientation by walls is supposed to be stronger than an aligning impact of C-dots in such conditions. The alignment of C_12_EO_4_ molecules perpendicular to PDMS walls is typical for the orientation of surfactant molecules in PDMS microchannels [2]. PDMS returns to its hydrophobic state after the plasma bonding of microchip elements, and the observed notable wall effects can be caused by intermolecular interactions of C_12_EO_4_ hydrocarbon tails with re-appearing CH_3_-groups that migrate from neighboring layers to internal microchannel surfaces and form the resulting hydrophobic internal surface of microchannels [65].

Thus, static LLC-bCD composite media demonstrated a higher-level molecular ordering in microfluidic confinement in addition to their intrinsic lamellar organization due to wall effects, heating–cooling cycles, presumable ordering around C-dots, and injection shear. At the next stage of this work, we continued analyzing the behavior of the LLC and composites by studying them in in dynamic microflows.

### 2.3. Impact of the Shear on the Molecular Arrangement in Microfluidic LLC and Composite Media

**Figure 5:**
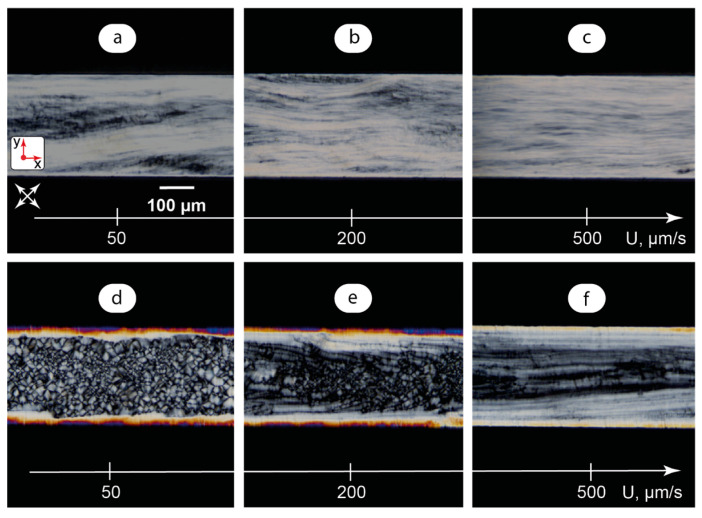
Dynamic samples of the LLC phase in a microchannel: (**a**–**c**) POM images of the LLC studied in dynamic conditions after pre-injection into the microchannel; (**d**–**f**) POM images of the LLC in a microchannel studied in dynamic conditions after heating up to the clearing point and further cooling down to 25 °C. Red arrows represent coordinate axes with respect to Figure 2. Crossed arrows indicate the positions of polarizers.

A unique advantage of microfluidic devices is their ability to create precisely tunable microflows of organized media. Therefore, this step of research focused on analyzing the orientation behavior of in-flow LLC-bCD systems at different flow velocities. Figure 5 summarizes the results.

The media were analyzed in the 0–1 mm/s average flow velocity range. The average flow velocity U was calculated from the flow rate set by the syringe pump: U = Q/(WH), where Q is the flow rate and W and H are the microchannel width and height, respectively. The term “average” is used to consider a non-uniform Poiseuille-like flow velocity profile in a microchannel that can be observed in a sheared non-Newtonian liquid crystal phase [26].

The optical behaviors of both the basic LLC and composites in dynamic conditions were found to be similar. In microchannels with a pre-injected phase of the basic LLC or composite (Figure 5a–c), an increase in the flow velocity led to a more uniform alignment of texture elements along a microchannel and a slight increase in transmittance that may indicate an additional shear-induced ordering of lamellae.

A considerably different flow behavior was demonstrated by the basic LLC or composite after the heating–cooling procedure (Figure 5d–f). The central lamellar texture with “onions” and wall-aligned sides were demonstrated to be resistant to slow microflows (Figure 5d) up to approximately 100 µm/s, so intermolecular interactions of LC molecules with PDMS walls or doped C-dots dominated over shear effects in this case. At the flow velocity of about 200 µm/s, the break-up of the texture started in the central part of the microchannel (Figure 5e), and a shear-induced realignment of “onions” and other lamellar patterns was observed. Microchannel sides were found to be more resistant to flow due to the decreasing flow velocity near the microchannel walls. Finally (Figure 5f), the shear-induced textures at 1 mm/s resembled those shown in Figure 5a, indicating a transition to the axial alignment of lamellae.

Higher-resolution images of the pure LLC and composite, taken at the 500× magnification and showing lamellar “onions” in more detail, are provided in Supplementary Materials. For the basic LLC and the composite injected into the microchannels at room temperature and dynamic systems, higher-resolution images revealed no significant additional details.

**Figure 6:**
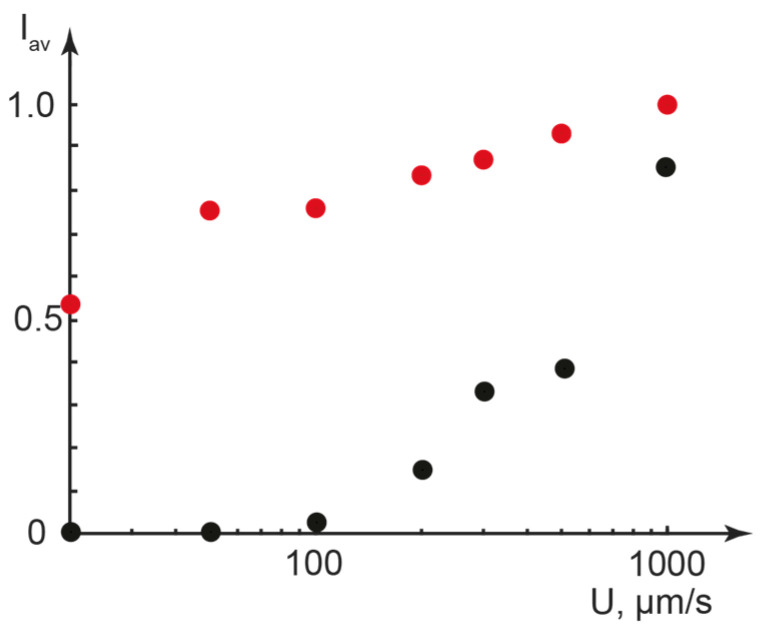
Normalized average transmission intensity of the mesophase vs. the flow velocity: red dots—dynamic samples after pre-injection into the microchannel; black dots—dynamic samples sheared after heating up to the clearing point and further cooling down to 25 °C.

To quantify the impact of the flow velocity on the molecular ordering in the basic LLC and composite flows and the resulting optical properties of these systems, we analyzed their POM images at different flow velocities with Equation (2). The images shown in Figure 5c,d were used as “max” and “min” references, respectively. Figure 6 demonstrates the results.

A slight increase in the transmittance of the unheated sample (Figure 6, red dots) is nearly linearly dependent on the logarithmic flow velocity. This is similar to the previously studied flow behavior of thermotropic nematic liquid crystals [47] and may indicate an additional flow-induced alignment of lamellar structures. The dynamic samples sheared after heating and subsequent cooling (Figure 6, black dots) show a plateau at flow velocities up to 100 µm/s, corresponding to maintaining a static intermolecular organization in the basic LLC or composite. Further increases in the average transmittance can be related to the break-up of this texture and a presumable rearrangement of lamellae along the flow.

In dynamic LLC and composite media, therefore, we can conveniently switch between various molecular organization states by applying the flow factor. Flow effects were also found to be reversible by applying an additional heating–cooling cycle, which returned these systems to their original oriented state.

### 2.4. Luminescence of Anisotropic LLC–Carbon Dot Systems in Confinement

**Figure 7:**
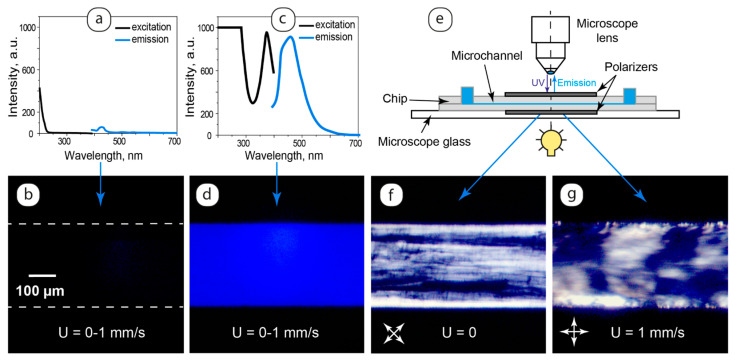
Luminescence of individual LLC and the composite in bulk and confinement: (**a**) the luminescence spectrum of the basic LLC; (**b**) the basic LLC in a microchannel in UV light; (**c**) the luminescence spectrum of the composite; (**d**) the composite in a microchannel in UV light; (**e**) microfluidic chip design for fluorescent microscopy imaging at crossed polarizers; (**f**,**g**) static and dynamic composites, respectively, at crossed polarizers and in UV light.

Combining C_12_EO_4_ lyotropic liquid crystal with luminescent carbon dots allowed for producing anisotropic molecular materials with intrinsic luminescent properties. The luminescent behavior of the composites was studied by fluorescence spectroscopy and microscopy. The results are summarized in Figure 7.

Emission from the basic LLC samples after their UV excitation was found to be negligible (Figure 7a). These properties were confirmed by fluorescent microscopy in microfluidic experiments performed with static and dynamic samples (Figure 7b).

The composite demonstrated blue light emission (Figure 7c) with the maximum at approximately 460 nm, which is close to the emission peak of carbon dot dispersions (Figure 1). Fluorescent microscopy images of the composite in the microchannel (Figure 7d) showed homogeneous blue light emission. Such uniform luminescence was observed in the entire studied flow velocity range of 0–1 mm/s both for the samples pre-injected into the microchannel and the samples studied after heating up to the clearing point and further cooling down to 25 °C. The orientation of LLC molecules at the microchannel walls also exerted no visible impact on composite luminescence at the microchannel sides.

Such luminescence behavior is different from the properties of a previously studied luminescent composite of a thermotropic nematic liquid crystal and quantum dots [47], where the luminescence of quantum dot clusters was restricted by inhomogeneities in the liquid crystal flow. The uniform luminescence of the LLC-bCD composite can be attributed to small additives of carbon dots (0.1 g/L) and their presumable uniform distribution in the composite. We can presume that the rearrangement of lamellae due to their alignment along the flow, interactions of the LLC molecules with microchannel walls, or the development of “onions” did not deteriorate the first-order intermolecular structure of the LLC phase represented by lamellar layers.

Uniform luminescence images of the composite (Figure 7d) did not allow for observing LLC textures in microchannels and monitoring the mobility or wall alignment of LLC in fluorescence microscopy experiments. To observe the LLC texture in such conditions, we modified microfluidic chips by placing them between two polarizing films arranged in a way to provide cross polarizers at different angles with respect to the microchannel axis (Figure 7e).

In Figure 7f,g, we can see LLC textures revealed by crossed polarizers in fluorescence microscopy experiments. In such microchips, the luminescence of carbon dots located in the oriented microfluidic LLC matrix can generate the resulting emission of polarized light. Such emission may be represented by blue light color in the microchannel images shown in Figure 7f,g.

In this work, therefore, we synthesized and analyzed LLC-bCD composites, which maintained stable and uniform luminescent properties at different flowrates and after pre-shear heating and cooling. Such composites are potentially capable of generating polarized luminescence from oriented LLC molecules in microchannels.

## 3. Discussion

The studied lyotropic liquid crystal systems and their hybrids with carbon dots were found to demonstrate an additional level of molecular ordering in microfluidic channels, which distinguishes them from previously studied confined thermotropic liquid crystal media and their hybrids with luminescent nanoparticles. The governing intermolecular interactions that provide such an organization are presumably interactions of amphiphilic LLC molecules with PDMS end groups on the microchannel surface and end groups of carbon dots along with the hydrophobic effect that is primarily responsible for the intrinsic lamellar organization of the basic LLC in aqueous media. The lamellar structure of LLC both in non-microfluidic conditions and in confinement can be considered as the first-level molecular ordering in such media. A combined impact of the microchannel walls, C-dots, and shear creates second-level ordering in confined composites, where lamellae can orient parallel to microchannel walls and bend at edges, re-arrange along the microchannel axis with the shear, or align around C-dots as multilayer onion-like structures. In microchannels, therefore, we observed a combination of nanoscale (lamellar layers) and interchangeable multiple microscale (additional lamellar ordering) structuring of the studied composites.

Carbon dots were found to exert an impact on the molecular ordering of LLC after cooling the composites from the isotropic liquid down to room temperature. In this case, added C-dots favored the formation of lamellar “onions” as possible defects around which such multilayer lamellar structures emerged. In pre-injected and dynamic composites and near microchannel walls, shear forces and wall effects dominated the ordering impact of carbon dots in the composite and the resulting molecular arrangement, as demonstrated by POM images.

The composites synthesized and characterized in this work represent a novel class of oriented luminescent molecular materials that highlight several trends of potential applications. First, the active response of LLC multilevel ordering towards the studied factors in a microchannel can be suitable for designing optically active microfluidic devices for photonic applications. Second, the composites represent hybrids with luminescent guest nanoparticles distributed in an aligned hosting LLC matrix. Such media are capable of generating polarized luminescence. This can generate multidisciplinary interest in employing such systems as bioanalytical tools for tracking and potentially quantifying biomarkers in lab-on-chip devices. Third, such hybrids can be models of biological anisotropic media in living capillary systems capable of incorporating and releasing biomolecules. Microfluidic confinement offers potential for the in-flow modeling and monitoring of these systems by simultaneously applying UV, visible, and polarized microscopy tools.

At this stage of studying LLC–carbon dot molecular systems, we focused on developing a composite, which maintains its anisotropic structure with doped luminescent nanoparticles and provides a stale and uniform luminescence. In addition to the confined orientation effects of the LLC matrix that were revealed in this work, future research directions can be logically highlighted. Varied additives of carbon dots will be tested to characterize the LLC phase existence range of the composite and its luminescent properties. Derivatives of ethylene glycol dodecyl ethers forming other types of mesophase, such as hexagonal, will be studied. Another future research focus will include the characterization of the studied system by electron microscopy, composite rheology studies, and an analysis of the impact of temperature on the molecular orientation of LLC molecules and the luminescence of composites in microfluidic confinement.

## 4. Materials and Methods

### 4.1. Materials

The lyotropic liquid crystal phase was produced from tetraethylene glycol monododecyl ether—C_12_H_25_(CH_2_CH_2_O)_4_OH or C_12_EO_4_ (Merck, Darmstadt, Germany), 99.999% purity, which was used as received. The LLC samples were prepared by mixing 45 w.% C_12_EO_4_, 5 w.% decanol, and 50 w.% water in glass vials. Small additions of decanol were used according to our previous work [50] to expand the concentration range of the lyotropic mesophase in the LLC systems formed by derivatives of ethylene glycol monododecyl ethers and continue experimental series with comparable results. LLC hybrids with carbon dots were prepared similarly to basic LLC media by using water with 0.1 g/L of pre-dispersed C-dots. All the samples were centrifuged at 3000 rpm (25 °C) for 30 min to perform the complete homogenization of the systems. Before further studies, all the fresh samples were kept at 25 °C for 14 days.

The carbon dots were synthesized by a hydrothermal method [51]. In total, 432 mg o-phenylenediamine and 840 mg citric acid monohydrate were dissolved in 80 mL deionized water, agitated with a magnetic stirrer for 10 min, and then autoclaved in a 100 mL Teflon autoclave at 180 °C for 9 h. The reaction product was cooled down and then centrifuged at 10,000 rpm for 10 min. The resulting solution was filtered with a 0.45 µm syringe filter. The filtered solution was further purified with the MW 1000 dialysis membrane (where MW stands for molecular weight) in water for 24 h, and the water was changed every hour. The synthesized carbon dots were dried and further used to prepare aqueous solutions with the required content of nanoparticles.

Microfluidic devices were fabricated from a polydimethylsiloxane (PDMS) Sylgard^TM^ 184 silicone elastomer (Dow Corning, Midland, MI, USA) and used as received. It came as a two-part elastomer kit (the pre-polymer and curing agent). The SU-8 3050 photoresist (Microchem Corp., Westborough, MA, USA) was used to produce a mold for microfluidic chips.

### 4.2. Methods

The orientation behavior of the LLC media in microfluidic flows was studied by polarized optical microscopy using an Olympus BX51 microscope (Olympus, Tokyo, Japan), equipped with a high-precision Linkam heating system that allows for providing uniform temperature conditions for experiments. Microscopy images were captured at 100× and 500× magnification using a ToupCam E3ISPM08300KPC camera (Touptek, Hangzhou, China).

The luminescent properties of the LLC-bCD composites in microchannels were studied by fluorescence microscopy using an Olympus BX43 fluorescent microscope (Olympus, Tokyo, Japan). Microscopy images were captured at 100× magnification using a ToupCam E3ISPM05000KPA camera (Touptek, Hangzhou, China).

Polarized microscopy images were processed by MATLAB 2021b software. The light transmission intensities were evaluated by processing polarized microscopy images using the pre-developed MATLAB script. For the comparison of light transmission intensities, all the respective microscopy images were taken at identical settings of both the microscope and the image-capturing software (ToupView, version 4.11) supplied with the camera.

The color profiles of the LLC at the microchannel edges were analyzed by comparing them with the Michel–Levy chart, considering the impact of image file color spaces and formats [63].

Hydrodynamic diameters of carbon dots were measured using a Malvern Zetasizer Nano ZS light scattering system (Malvern Instruments Ltd, Worcestershire, United Kingdom). The reported diameters of the particles corresponded to the maximums of the distribution curves provided by Malvern Zetasizer Nano ZS version 7.13 software reports.

The photoluminescence emission spectra of the pure LLC and composite were recorded by a Varian Cary Eclipse spectrofluorimeter (Agilent, Santa Clara, CA, USA).

### 4.3. Fabricating Microfluidic Devices and Preparing the Experimentsal Setup

Microfluidic devices were fabricated using standard photolithography techniques [66]. The chips with rectangular microchannels were produced using this technology. The length, width, and height of all the microchannels were 15 mm, 300 μm, and 100 μm, respectively. An SU-8 photoresist and a transparent photomask with a negative image of a microchip were used to produce a 100 µm-thick mold of microfluidic chips on top of a 3-inch silicon wafer. PDMS pre-polymer was mixed with a curing agent, poured over the mold, and allowed to cure in an oven for 4 h at 60 °C. Once cured, PDMS was peeled off the mold and bonded to a flat PDMS slab via 1 min plasma treatment using the Harrick Plasma Cleaner PDC-23G (Harrick Plasma, Ithaca, NY, USA). The PDMS device was then heated in an oven at 180 °C for 1 h to complete the bonding of the two polymer layers.

The basic LLC and composite samples were infused into microfluidic devices using Shenchen ISPLab01 syringe pumps (Baoding Shenchen Precision Pump Co., Ltd., Baoding city, China). In this work, the flow velocities were varied in the range of 0.05–1 mm/s. All the static and dynamic microfluidic experiments were performed at 25 °C.

To provide the same hydraulic paths for all the fluids, PTFE tubes of identical lengths (10 cm) and internal diameters that fit the same needle tips inserted into the microchip outputs (20 G-type needles, 0.9 mm diameter) were used. These tubes were connected to identical 1 mL syringes used for infusing the individual LLC or composite by the syringe pump.

## 5. Conclusions

The microchannel wall effects, phase transitions, and shear in the velocity range up to 1 mm/s were shown to be selective factors that added a higher-level degree of ordering to an intrinsic lamellar self-organization of the studied LLC–carbon dots composite.

In a pre-infused composite or pure LLC, a predominant axial arrangement of lamellae was observed, which increased with the shear according to the POM studies. Cooling the composite from an isotropic liquid to 25 °C resulted in a homeotropic orientation of LC molecules near the microchannel walls. Doped carbon dots created a distinctively ordered structure as presumable centers for 20–40 µm onions in central parts of the microchannels after cooling from isotropic liquid, as compared with the basic LLC. Such structures resisted flow velocities up to 100–200 µm/s and then gradually transformed into a predominant axial orientation of lamellae. Such an ordering provided a quantitatively evaluated change in the transmission intensity of the microchannel media and, on the other hand, maintained the intensive and homogeneous luminescence of the composite in the range of applied microfluidic factors.

The discussed molecular behavior of the studied LLC–carbon dot composites highlights potential applications for these new luminescent materials as in-flow structuring agents, models of biological anisotropic media, and sensors integrated into lab-on-chip or organ-on-chip platforms.

## Data Availability

Data are contained within the article and Supplementary Materials.

## Supplementary Materials

The supporting information can be downloaded at: https://www.mdpi.com/article/10.3390/ijms25105520/s1.
