# Supplementary material for: Molecular Orientation Behavior of Lyotropic Liquid Crystal–Carbon Dot Hybrids in Microfluidic Confinement
Source: Int J Mol Sci. 2024 May 18;25(10):5520. doi: 10.3390/ijms25105520 (PMC11122583; doi:10.3390/ijms25105520)
Supplement: Supplementary file 1 [file ijms-25-05520-s001.zip › ijms-2979081-supplementary.pdf]

Optical Properties of Liquid Crystal and C-dots

UV-Vis spectroscopy data and photoluminescence (excitation and emission) of the liquid crystal and C-dots are given in Figures S1 and S2.

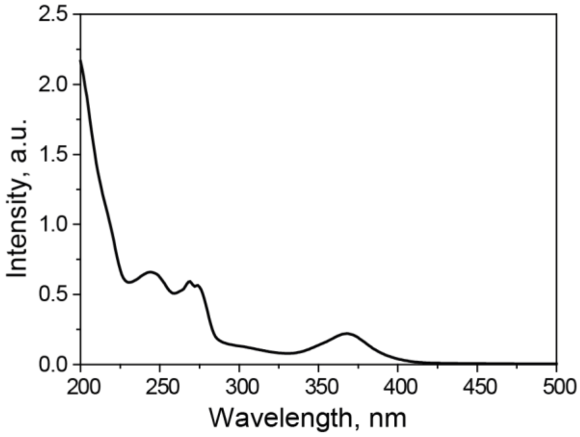

Figure S1. UV-Vis absorption of bCD in water (concentration 0,01 mg/ml).

We can see in in Figure S1 that the absorption maximum of C-dots is observed at 365 nm. C-dots absorb UV light and generate emission in the visible light range. Liquid crystal itself possesses negligible luminescence properties. The results of the liquid crystal and C-dots luminescence studies are shown in Figure S2.

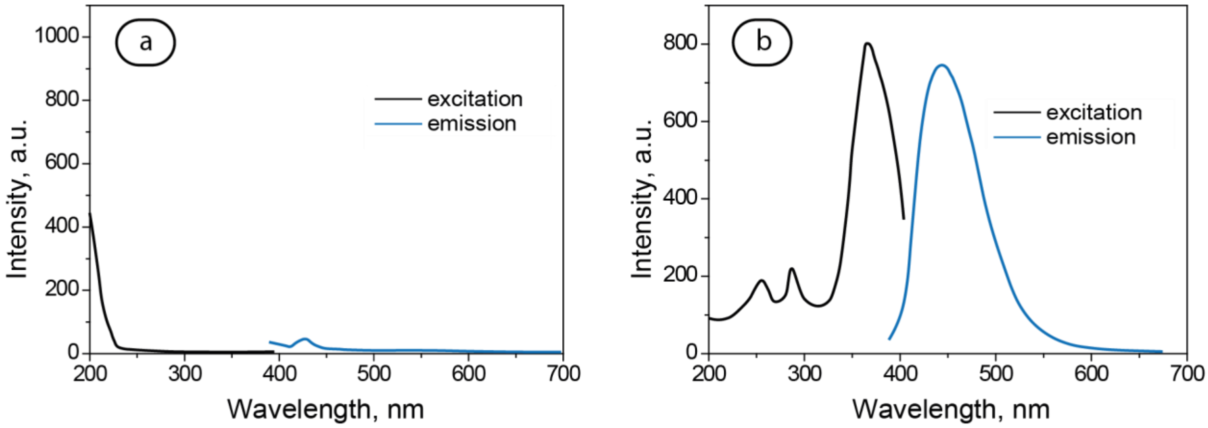

Figure S2. Photoluminescence (excitation and emission) of the liquid crystal (a) and C-dots (b).

We can see in Figure S2 that intensive luminescence of C-dots initiated by absorption of UV light makes them an optically active (luminescent) component of their composite with the lyotropic liquid crystal.

Evaluation of the size of C-dots

The results of dynamic light scattering experiments demonstrating the presence of nanoscale particles in the analyzed samples of synthesized C-dots are shown in Figure S3.

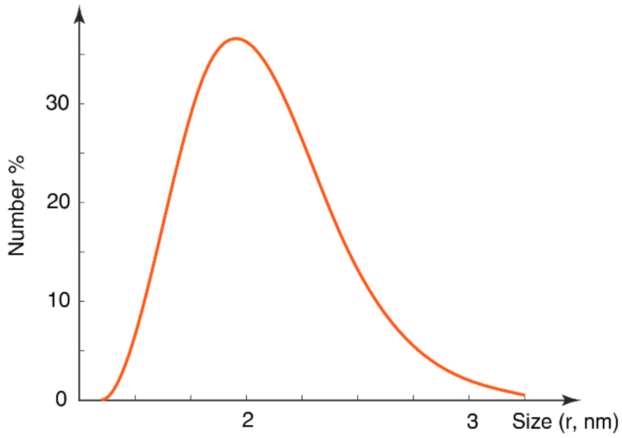

Figure S3. Hydrodynamic radius of C-dots according to dynamic light scattering experiments.

Figure S3 demonstrates the results provided by Malvern Zeta Sizer Nano. According to Figure S3, the studied sample of carbon dots contains nanoparticles with the size of several nanometers.

High Resolution Images of the Liquid Crystal and Composite

For a better clarity, higher resolution images of the liquid crystal and composite in microchannels were taken at 500x magnification. The results are shown in Figure S4.

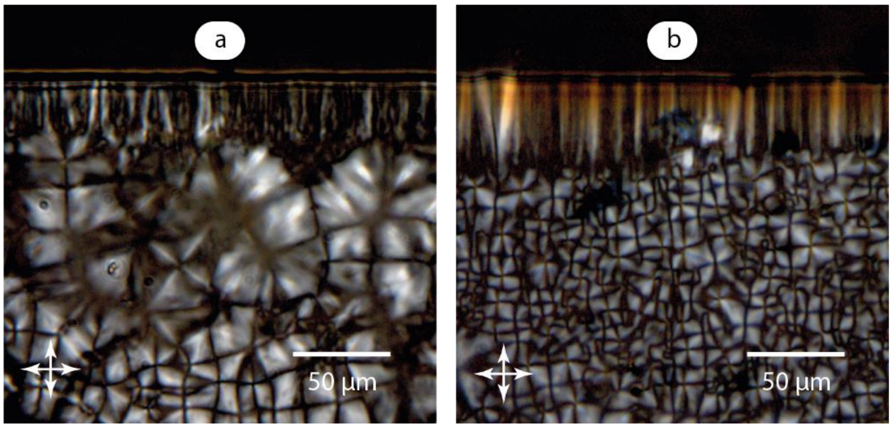

Figure S4. Comparative microscopy images of the liquid crystal (a) and composite (b) after heating to the isotropic liquid state and further cooling down to room temperature.

Comparison of textures demonstrates smaller, denser, and more uniform “onion” structures in the composite as compared with pure LLC.
